# Improving grazing-incidence small-angle X-ray scattering–computed tomography images by total variation minimization

**DOI:** 10.1107/S1600576719016558

**Published:** 2020-02-01

**Authors:** Hiroki Ogawa, Shunsuke Ono, Yukihiro Nishikawa, Akihiko Fujiwara, Taizo Kabe, Mikihito Takenaka

**Affiliations:** a Institute for Chemical Research, Kyoto University, Gokasho, Uji, Kyoto 611-0011, Japan; b JST, PRESTO, 4-1-8 Honcho, Kawaguchi, Saitama 332-001, Japan; c Riken SPring-8 Center, 1-1-1 Kouto, Sayo, Hyogo 679-5148, Japan; d School of Computing, Tokyo Institute of Technology, 4259 Nagatsuta-cho, Midori-ku, Yokohama, Kanagawa 226-8503, Japan; e Department of Macromolecular Science and Engineering, Kyoto Institute of Technology, Kyoto 606-8585, Japan; f Department of Nanotechnology for Sustainable Energy, Kwansei Gakuin University, Hyogo 669-1337, Japan; g Japan Synchrotron Radiation Research Institute, 1-1-1 Kouto, Sayo, Hyogo 679-5198, Japan

**Keywords:** grazing-incidence small-angle scattering, X-ray tomography, diffuse scattering, surface structure, soft matter

## Abstract

Grazing-incidence small-angle X-ray scattering (GISAXS) coupled with computed tomography (CT) has enabled the visualization of the spatial distribution of nanostructures in thin films. 2D GISAXS images are obtained by scanning along the direction perpendicular to the X-ray beam at each rotation angle. Because the intensities at the *q* positions contain nanostructural information, the reconstructed CT images individually represent the spatial distributions of this information (*e.g.* size, shape, surface, characteristic length). These images are reconstructed from the intensities acquired at angular intervals over 180°, but the total measurement time is prolonged. This increase in the radiation dosage can cause damage to the sample. One way to reduce the overall measurement time is to perform a scanning GISAXS measurement along the direction perpendicular to the X-ray beam with a limited interval angle. Using filtered back-projection (FBP), CT images are reconstructed from sinograms with limited interval angles from 3 to 48° (FBP-CT images). However, these images are blurred and have a low image quality. In this study, to optimize the CT image quality, total variation (TV) regularization is introduced to minimize sinogram image noise and artifacts. It is proposed that the TV method can be applied to downsampling of sinograms in order to improve the CT images in comparison with the FBP-CT images.

## Abstract 2

Grazing-incidence small-angle X-ray scattering coupled with computed tomography (CT) has enabled the visualization of the spatial distribution of nanostructures in thin films. In this study, to optimize the CT image quality, total variation regularization is introduced to minimize sinogram image noise and artifacts.

## 1. Introduction   

Grazing-incidence small-angle X-ray scattering (GISAXS) is widely used to characterize the nanostructural features of metallic and polymer materials in thin films (Lee *et al.*, 2005 ▸; Liu *et al.*, 2015 ▸; Kaune *et al.*, 2009 ▸). Because of the high intensity and small beam size of the incident X-rays of synchrotron radiation (SR), GISAXS has extended to scanning measurements occurring over a short time period (Hexemer & Müller-Buschbaum, 2015 ▸; Saito *et al.*, 2015 ▸; Lu *et al.*, 2013 ▸; Gann *et al.*, 2014 ▸). Recently, GISAXS coupled with the computed tomography (CT) method was successfully used to visualize the spatial distribution of metallic nanoparticles on substrates (Kuhlmann *et al.*, 2009 ▸; Ogawa *et al.*, 2015 ▸, 2017 ▸).

In the conventional CT method, CT images are reconstructed from sinograms of the absorption intensities taken from different angles through a sample (Herman, 1980 ▸; Elbakri & Fessler, 2002 ▸; Chen *et al.*, 2008 ▸; Nishikawa *et al.*, 2012 ▸). In the GISAXS-CT method, CT images are reconstructed from sinograms obtained by scanning GISAXS measurements along the direction (*Y*) perpendicular to the X-ray beam at each rotation angle (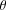). In GISAXS images, information on the nanometre scale in reciprocal space is contained in the scattering intensities at the *q* positions. Since CT images are reconstructed from the sinograms of the scattering intensities, one can obtain the spatial distributions of structural information corresponding to the scattering intensities. This technique has been applied to transmission small-angle X-ray scattering (SAXS)-CT as well as GISAXS-CT methods (Schroer *et al.*, 2006 ▸; Schaff *et al.*, 2015 ▸; Skjønsfjell *et al.*, 2016 ▸; Liebi *et al.*, 2018 ▸). In these methods, CT images are reconstructed from the sinograms using filtered back-projection (FBP). We refer to these reconstructed CT images as FBP-CT images.

To obtain a high-quality FBP-CT image, an enormous number of scattering images are required. In addition, the sampling rate of the rotation angles must satisfy the Shannon/Nyquist sampling theorem (Candès *et al.*, 2006 ▸; Khan & Chaudhuri, 2014 ▸). If GISAXS measurements with a step size of 20 µm along the *Y* direction at each rotation angle are performed on a target sample of size 1.0 mm, then the number of rotation angle steps must be greater than 79. In this case, the total number of scattering images is more than 3950. This means that even if one can acquire GISAXS data in 1 s using SR, the total time required for measurement is more than 1 h. Experiments using SR need to be carried out within a limited time period; thus, long measurement times can decrease the number of samples that can be measured. Moreover, high-quality FBP-CT images require sampling with high X-ray radiation doses, but soft-material samples are sensitive to X-ray-induced radiation damage. Thus, an approach for low dose and high speed is indispensable for the GISAXS-CT method.

Recently, Hu and co-workers proposed a technique for generating projection images from limited-angle SAXS data, using the ordered subset expectation maximization (OSEM) method (Hu *et al.*, 2017 ▸; Hudson & Larkin, 1994 ▸). These researchers showed that the OSEM algorithm could effectively eliminate streaking artifacts and improve the efficiency of data acquisition by at least three times compared with the FBP algorithm. To further improve the CT images, a more effective framework is required for image reconstruction from very limited angle (grazing-incidence) SAXS data.

In this article, we propose a new image reconstruction framework for GISAXS-CT. Besides the FBP algorithm, many efforts have been made to develop iterative CT algorithms, which are all the derivatives of fitting algorithms. Those efforts can be roughly classified into two kinds: one is to improve the robustness of the fitting, and the other is to optimize the calculation efficiency. This article is mainly focused on the former issue. In the case of OSEM, the ordered subset (OS) mainly concerns the calculation efficiency by effectively reducing the data set (projection) in a single fitting loop. The expectation maximization (EM) enhances the robustness of the fitting. Our framework is realized as a nonlinear filtering algorithm that can be used instead of existing reconstruction methods, such as the FBP and the OSEM algorithms. The nonlinear filter is built upon a constrained optimization problem involving total variation (TV) regularization (Rudin *et al.*, 1992 ▸). TV regularization is a mathematical model that characterizes 2D ‘piecewise-smooth’ signals and has been shown to be a powerful technique for image denoising and decomposition tasks (*e.g*. Chambolle, 2004 ▸; Ono *et al.*, 2014 ▸). Our framework based on TV regularization enhances the robustness of the fitting rather efficiently by incorporating the recent progress in the field of optimization problems. The regularization term used in our framework enables the reduction of the input data set while keeping the quality of the resulting images. This approach is also expected to help compensate for the incompleteness of the data set, for example, the absence of particular angular regions in the projection series, termed ‘missing wedges’, and opaque (no transmission) regions, for example ‘metal artifacts’ (Arslan *et al.*, 2006 ▸; Bamberg *et al.*, 2011 ▸). However, we think that these matters should be discussed elsewhere. Our framework enables high-quality reconstruction from the sinograms obtained by scanning GISAXS measurements with limited interval angles from 3 to 48°. We also discuss the possibility of using our framework as a low-dose and high-speed approach by comparing images reconstructed by our framework (hereafter called TV-CT images) and FBP-CT images.

## 2. Experiment   

### 2.1. Sample   

We prepared thin circular Au layers on a silicon (Si) substrate for the GISAXS-CT measurements; these layers were deposited using the plasma sputter-coating method. Sputtering was accomplished using Ar ion beams and Au targets (ESC-101, ELIONIX). To form circular layers of 1 mm in diameter, we created circular through-holes in a 0.5 mm-thick aluminium mask using a drilling device. Au was sputtered onto the substrate for 1400 s, resulting in Au-patterned layers that were ∼100 nm thick. Defects were produced on a part of the circular pattern by scratching with tweezers.

### 2.2. GISAXS-CT measurements and optical microscopy observations   

GISAXS measurements were performed at the first experimental hutch of the beamline BL03XU, Advanced Soft Matter Beamline (FSBL), at SPring-8. The hutch is dedicated to GISAXS experiments using an intense beam (10^13^ photons s^−1^) with very low divergence [12.3 µrad (horizontal) × 1.1 µrad (vertical)]. The X-ray wavelength λ and the sample–detector distance were 0.1 nm and 2275 mm, respectively (Ogawa *et al.*, 2013 ▸). The full width at half-maximum (FWHM) of the beam at the sample position was 28.5 µm (horizontal) × 99.5 µm (vertical), and the beam footprint (which could entirely cover the pattern configurations) was extended to 11.4 mm at an incidence angle of 0.50°. The scattering images were detected by a PILATUS 1M (Dectris Ltd) with an exposure time of 1.0 s.

**Figure 1:**
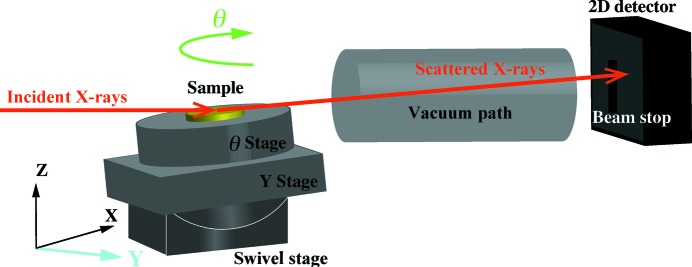
A schematic sketch of the experimental setup of GISAXS-CT.

To reconstruct the images in the lateral directions using the CT method, we scanned the sample over a distance spanning 1.02 mm in steps of 15.0 µm, along the direction normal to the incident beam (*Y* direction). The scanning steps were comparable to the incident beam’s width (the beam’s size in the horizontal direction). In the rotation scan (θ), images were acquired in 1.0° steps for 0.0 ≤ θ < 180.0°. A diagram of the experimental setup is shown in Fig. 1 ▸.

An optical microscopy (OM) image of the fabricated Au-patterned thin layer on the substrate was acquired using a digital microscope (VHX-5500, KEYENCE).

### 2.3. TV minimization   

In this section, we establish a new image reconstruction framework for GISAXS-CT. First, we introduce a measurement model for GISAX-CT as follows:
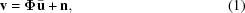
where 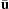 is the original CT image we wish to reconstruct, **v** is a sinogram image, 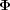 is a matrix representing the GISAX-CT measurement process, possibly with limited interval angles, and **n** is the measurement noise. We note that the images 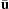 and 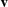 in the model are treated not as 2D matrices but as 1D vectors for mathematical convenience. Both treatments are essentially the same, and in our implementation the images are processed as is (not vectorized).

Under this model, our framework is formalized as a constrained optimization problem as follows: find 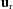 that minimizes 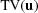 subject to 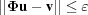, where 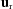 is the reconstructed CT image, the objective function 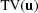 is the TV regularization and the constraint 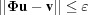 is an L2 data-fidelity criterion. Here, the parameter 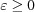 controls the ‘reliability’ of the observed sinogram 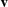, that is, the smaller 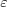 is, the smaller is the estimated noise contamination of the sinogram. Moreover, minimizing 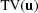 means that the reconstructed CT image 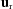 should be ‘piecewise-smooth’; that is, the TV regularization prefers artifact-free images. After some reformulations, we can solve this problem by a state-of-the-art convex optimization algorithm, called the primal-dual splitting method (Condat, 2013 ▸), yielding an efficient algorithmic framework for reconstructing GISAXS-CT images.

We attempt to give an intuitive explanation of our problem formulation. For simplicity, here we assume the noiseless case, that is, 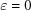, and the constraint is reduced to a linear equation 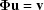. If the interval angles are limited, then this linear equation becomes underdetermined, meaning that there are infinitely many solutions to the linear equation. Thus, we need some criterion to characterize a reasonable solution (a reconstructed image) among all possible solutions. In our problem formulation, TV regularization plays a role. More specifically, minimizing 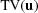 subject to 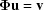 implies picking out a piecewise-smooth image from the possible reconstructed CT images that satisfy the linear equation. As a result, we can reconstruct a reasonable CT image under scenarios of GISAXS data with limited interval angles. We refer to these reconstructed CT images as TV-CT images. The TV regularization algorithm roughly consists of four computations: taking neighboring differences, a soft-thresholding operation, matrix–vector multiplication with **Φ** and metric projection onto an L2-norm ball.

## 3. Results and discussion   

**Figure 2:**
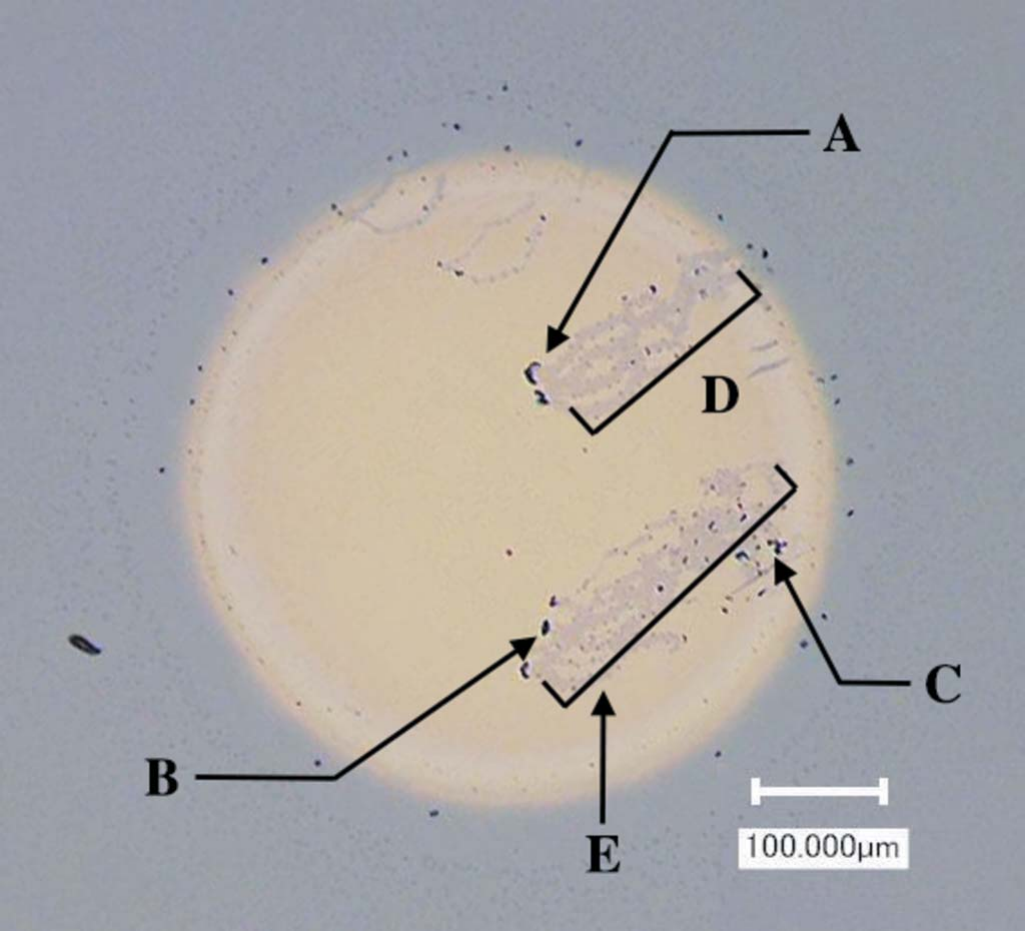
Optical microscopy image of the measured sample. A circular pattern was deposited by Au sputtering.

**Figure 3:**
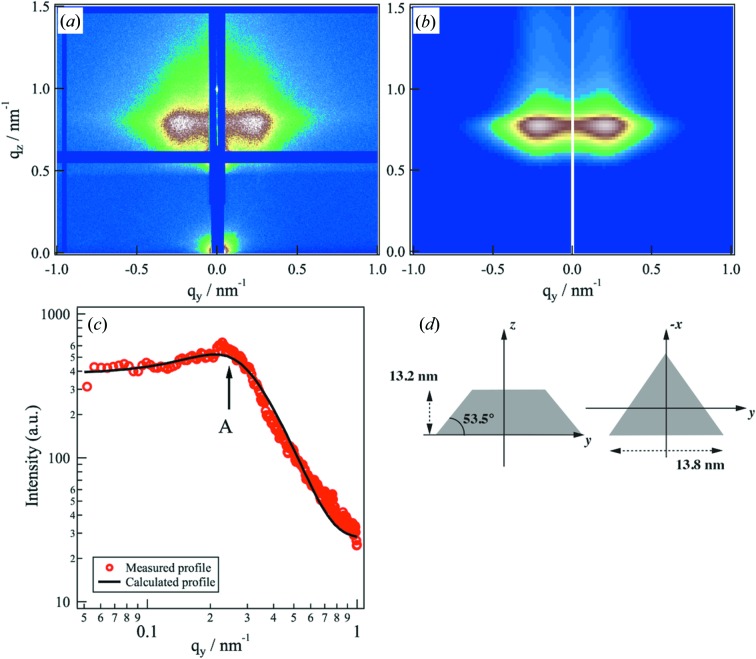
(*a*) Measured and (*b*) calculated 2D GISAXS images from a circular pattern of Au nanoparticles on an Si substrate at an incidence angle of 0.50°. (*c*) The in-plane profile obtained from the measured 2D pattern at *q_z_* = 0.80 nm^−1^ (red circles) and the calculated profile (black solid line). The arrow indicates the position at which the CT image was reconstructed from the intensities. (*d*) A cartoon of the structural model obtained from the calculation result.

Fig. 2 ▸ shows an OM image of a thin circular Au layer on an Si substrate. We observed impurities at parts A, B and C of the circular pattern. In addition to these impurities, the OM image exhibited different colors in regions D and E, indicating that the thin Au film contained scratched areas. We acquired a GISAXS pattern of the area free from defects by irradiating the center position of the circle. Fig. 3 ▸(*a*) shows a 2D GISAXS image from the thin Au film at an incidence angle of 0.50°. The scattering peaks were observed at (*q_y_*, *q_z_*) = (±0.24, 0.80 nm^−1^), indicating that periodicity exists in the thin Au layer. To characterize the morphology of the thin Au layer, we adopted a truncated tetrahedron with periodicity. Fig. 3 ▸(*b*) shows the 2D calculated scattering pattern. We used the *FitGISAXS* software for the analysis of the GISAXS data within the distorted-wave Born approximation (Babonneau, 2010 ▸). For modeling, we used a truncated tetrahedron form factor and the Percus–Yevick 2D model with a Gaussian distribution function as the structure factor. Fig. 3 ▸(*c*) shows the measured and calculated in-plane intensity profiles at *q*_*z*_ = 0.80 nm^−1^. The in-plane profile from modeling was in good agreement with the measured in-plane profile. Fig. 3 ▸(*d*) shows a cartoon illustrating the structural model. The calculated result demonstrated that the mean triangle length was 13.8 nm and the height was 13.2 nm, and the angle between the triangle face and the height was 53.5°. Additionally, the mean particle distance between the truncated tetrahedra was 18.0 nm. The appearance of a broad peak at *q_y_* = 0.24 nm^−1^ reflected the interparticle distance between the Au nanoparticles. We note that our model did not perfectly match with the vertical profile for the higher *q_z_* region above *q_z_* = 0.92 nm^−1^. This may be the effect of inhomogeneous surface roughness or the film thickness in the irradiated part of the sample.

**Figure 4:**
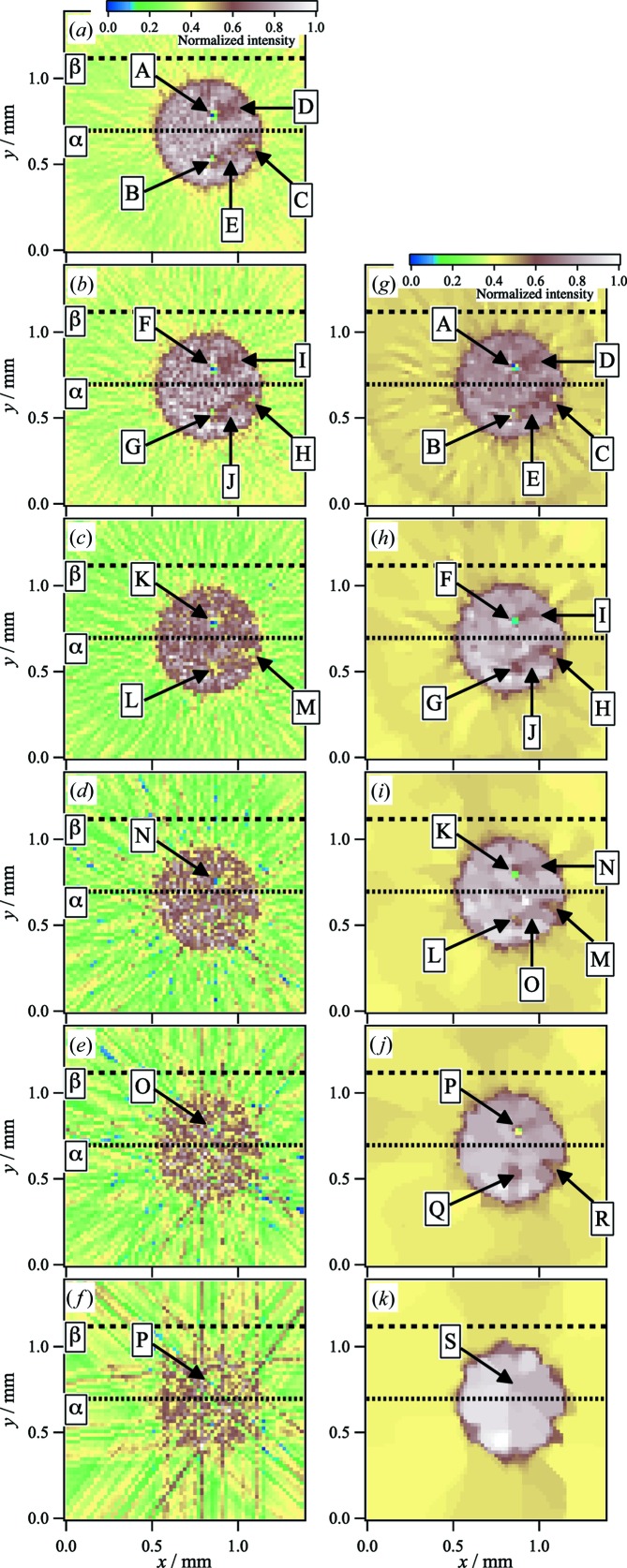
(Left) Reconstructed CT images as a function of Δθ. (*a*) Δθ = 1.0°; (*b*) Δθ = 3.0°; (*c*) Δθ = 6.0°; (*d*) Δθ = 12.0°; (*e*) Δθ = 24.0°; and (*f*) Δθ = 48.0°. The CT images were obtained from projections at the *q_y_* positions of 0.24 nm^−1^ in the in-plane profiles at *q_z_* = 0.80 nm^−1^. (Right) Recovered CT images using TV regularization for downsampling CT images: (*g*) Δθ = 3.0°; (*h*) Δθ = 6.0°; (*i*) Δθ = 12.0°; (*j*) Δθ = 24.0°; and (*k*) Δθ = 48.0°.

Fig. 4 ▸(*a*) shows the FBP-CT image reconstructed from the sinograms using the intensities at the *q_y_* position of 0.24 nm^−1^ [as indicated by A in Fig. 3 ▸(*c*)]. We note that all intensity values in the CT image were divided by the maximum intensity to obtain the normalized intensities. When we selected the intensities for the first scattering peak at *q_y_* = 0.24 nm^−1^, the FBP-CT image exhibited a circular pattern. Inside the pattern, we observed lower intensities around regions A–C in Fig. 4 ▸(*a*). The OM image showed impurities in the corresponding areas. We also observed scratched areas as weak-intensity areas in regions D and E. This result indicated that there is a lower density of Au nanoparticles in these scratched areas. The resolution of the obtained images of the impurities and scratched areas is lower than that of the corresponding OM image. For example, three lines can be observed in region D in Fig. 2 ▸, while only smeared images of the lines are observed in the FBP-CT image. This is because the spatial resolution of the reconstructed image was limited to FWHM = 28.5 µm by the incident beam’s width.

For the data acquisition process, we scanned a distance spanning 1.02 mm with steps of 15.0 µm along the *Y* direction. According to the Shannon/Nyquist sampling theorem, the number of angle views to perfectly reconstruct images must be over 40 points. In this reconstruction process, angular views of 180 points satisfied this theorem. Hence, we assumed that the FBP-CT image acquired with an interval angle (Δθ) of 1.0° step for 0.0 ≤ θ < 180.0° in Fig. 4 ▸(*a*) was the ‘true image’. In Figs. 4 ▸(*b*)–4 ▸(*f*), we show the changes in the FBP-CT images with Δθ. In the case of Δθ = 3.0° shown in Fig. 4 ▸(*b*), we could identify both the impurities and scratched parts (as indicated by F–J). In the FBP-CT image of the Δθ = 6.0° step in Fig. 4 ▸(*c*), although impurities were visible at K, L and M, it was difficult to identify the scratched areas. When Δθ was increased to 12.0°, the higher noise made it difficult to determine the impurities and scratched areas, as shown in Fig. 4 ▸(*d*). As shown in Figs. 4 ▸(*e*) and 4 ▸(*f*), the FBP-CT images with Δθ = 24.0° and Δθ = 48.0°, respectively, suffered significantly from the higher noise, and it was difficult to identify the circle pattern and the defect areas.

Figs. 4 ▸(*g*)–4 ▸(*k*) show the CT images obtained by our framework (TV-CT images). Compared with the FBP-CT images, the TV-CT images are much improved. In the TV-CT image with Δθ = 3.0° step, the noise in the circular pattern and the artifacts in the background area were suppressed by TV regularization, and the impurities (indicated by A–C) and scratched regions (indicated by D and E) were preserved in Fig. 4 ▸(*g*), in contrast to the FBP-CT images with the same Δθ = 3.0° step shown in Fig. 4 ▸(*b*). For the Δθ = 6.0° step, the intensities of the noise in the circular pattern and the artifacts were suppressed, and the intensities in the circular patterned area were enhanced. Hence, we could confirm that the impurities at F–H and scratched regions at I and J were preserved, as shown in Fig. 4 ▸(*h*). In the case of Δθ = 12.0°, although it was difficult to identify the impurities and scratched parts in the FBP-CT image in Fig. 4 ▸(*d*), the image quality was improved. In the TV-CT image in Fig. 4 ▸(*i*), the impurities became visible at K–M. Moreover, it was surprising that the scratched parts appeared around the region indicated by N and O in Fig. 4 ▸(*i*). The apparent artifacts were also clearly suppressed by TV regularization. For the Δθ = 24.0° step, since the FBP-CT image showed significantly increased noise and artifacts, the circular pattern was blurred, as shown in Fig. 4 ▸(*e*). By using TV regularization, the denoising and edge preservation effects led to the recovery of the circular pattern in Fig. 4 ▸(*j*). Moreover, impurities were visible (as indicated by P–R). For the Δθ = 48.0° step, owing to the apparent artifacts, we could not identify the shape of the circle in the FBP-CT image [Fig. 4 ▸(*f*)]. In Fig. 4 ▸(*k*), the TV-CT image showed the shape of the circle, although the edges were blurred. Because of the larger interval angle, the artifacts were more pronounced in the circular pattern. When TV regularization removed the apparent noise, the algorithm overly smoothed the circular pattern so that the impurities were not visible (as indicated by S).

**Figure 5:**
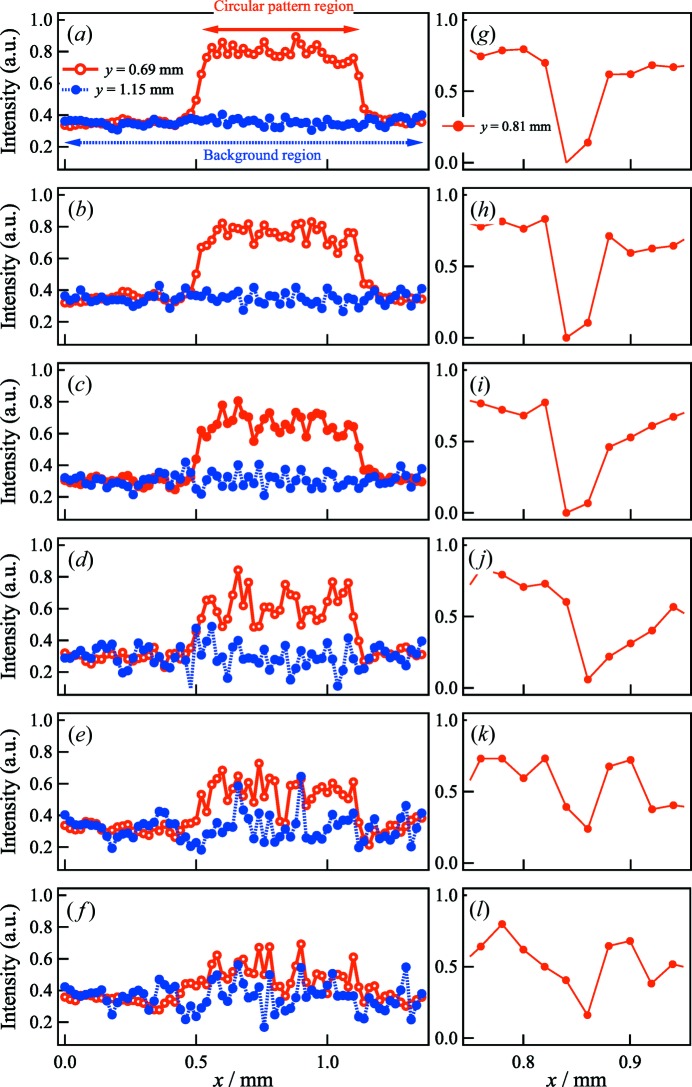
(Left) Cross-sectional normalized intensity profiles at *y* = 0.69 mm and *y* = 1.15 mm (along α and β) in Figs. 4 ▸(*a*)–4 ▸(*f*): (*a*) Δθ = 1.0°; (*b*) Δθ = 3.0°; (*c*) Δθ = 6.0°; (*d*) Δθ = 12.0°; (*e*) Δθ = 24.0°; and (*f*) Δθ = 48.0°. (Right) Cross-sectional normalized intensity profiles at *y* = 0.81 mm through A, F, K, N, O and P in Figs. 4 ▸(*a*)–4 ▸(*f*). (*g*) Δθ = 1.0°; (*h*) Δθ = 3.0°; (*i*) Δθ = 6.0°; (*j*) Δθ = 12.0°; (*k*) Δθ = 24.0°; and (*l*) Δθ = 48.0°.

The increase in noise with Δθ can be clearly observed in the intensity profile of the FBP-CT images. The cross-sectional normalized intensity profiles for *y* = 0.69 mm in Figs. 4 ▸(*a*)–4 ▸(*f*) [as indicated by the dotted line α in Figs. 4 ▸(*a*)–4 ▸(*f*)] are plotted as a function of *x* in Figs. 5 ▸(*a*)–5 ▸(*f*), respectively. The intensities for the circle pattern decreased as Δθ increased, and the noise increased. We also plotted the profiles for *y* = 1.15 mm, corresponding to the background area, in Figs. 5 ▸(*a*)–5 ▸(*f*) [as indicated by the dotted line β in Figs. 4 ▸(*a*)–4 ▸(*f*)]. The intensities of the artifacts in the background areas also increased with Δθ. In Figs. 5 ▸(*g*)–5 ▸(*l*), we plotted the profiles for *y* = 0.81 mm as a function of *x* at the location of the impurity [as indicated by A, F, K, N, O and P in Figs. 4 ▸(*a*)–4 ▸(*f*), respectively]. We were able to confirm the presence of impurities for Δθ ≤ 6.0° in Figs. 5 ▸(*g*)–5 ▸(*i*). When Δθ increased to 12.0°, the noise increased around the impurities [Fig. 5 ▸(*j*)]. For the profiles of Δθ = 24.0° and Δθ = 48.0°, the higher noise made it difficult to identify the impurities in Figs. 5 ▸(*k*) and 5 ▸(*l*), respectively.

**Figure 6:**
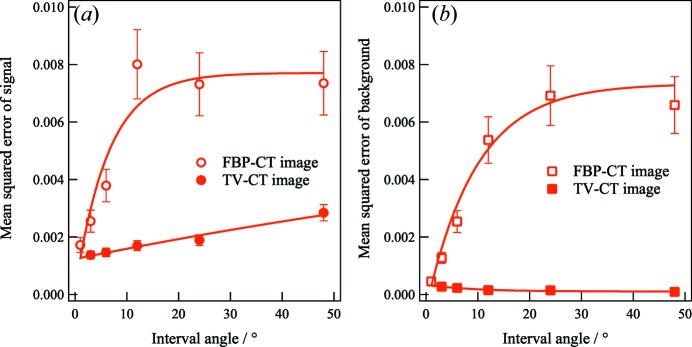
MSE values as a function of Δθ in (*a*) the circular patterned and (*b*) background areas obtained from Figs. 4 and 7. Open circles and squares were estimated from the line profiles of FBP-CT images with various Δθ angles. Filled circles and squares were estimated from the line profiles of the TV-CT images using TV regularization.

To evaluate the image quality for the circular pattern and background regions, we plotted the mean squared error (MSE) as a function of Δθ in Figs. 6 ▸(*a*) and 6 ▸(*b*). The MSE is written as
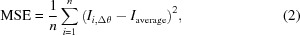
where *i* represents several data points for the circular pattern or background regions along the *x* direction. 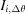 denotes the normalized intensity of a point *i* at each interval angle 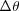. We used the intensity values for the circular pattern region between *x* = 0.54 mm and *x* = 1.10 mm and the background region between *x* = 0.00 mm and *x* = 1.36 mm, which are shown in Figs. 5 ▸(*a*)–5 ▸(*f*). 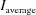 represents the averaged intensities of the circular pattern or background regions.

For the true image, the MSE of the circular pattern was estimated to be 0.00172 in Fig. 6 ▸(*a*). For the Δθ = 12.0° step, this value was drastically increased to 0.00881. For Δθ ≥ 12.0°, the MSE values were almost constant, although 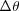 increased (as indicated by red circles). The errors are determined by the noise in the CT images. Since the noise increased with increasing values of Δθ, the errors increased as a function of Δθ. However, the ramp filter in the FBP process may reduce higher noise in the signal for θ ≥ 12.0°, resulting in saturation of the MSE value for Δθ ≥ 12.0°.

In Fig. 6 ▸(*b*), we show the MSE of the background area as a function of 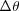 (as indicated by red squares). For the lowest intensities of the artifacts in the true FBP-CT image, the MSE of the area was estimated to be 0.00046. As the interval angles increased, an increase in the intensities of artifacts led to an increase in the MSE values until 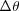 = 12.0°. The value from the Δθ = 12.0° step was approximately 14 times larger than that from the Δθ = 1.0° step. As described above, the MSE values for the background area were almost constant for Δθ ≥ 12.0°.

**Figure 7:**
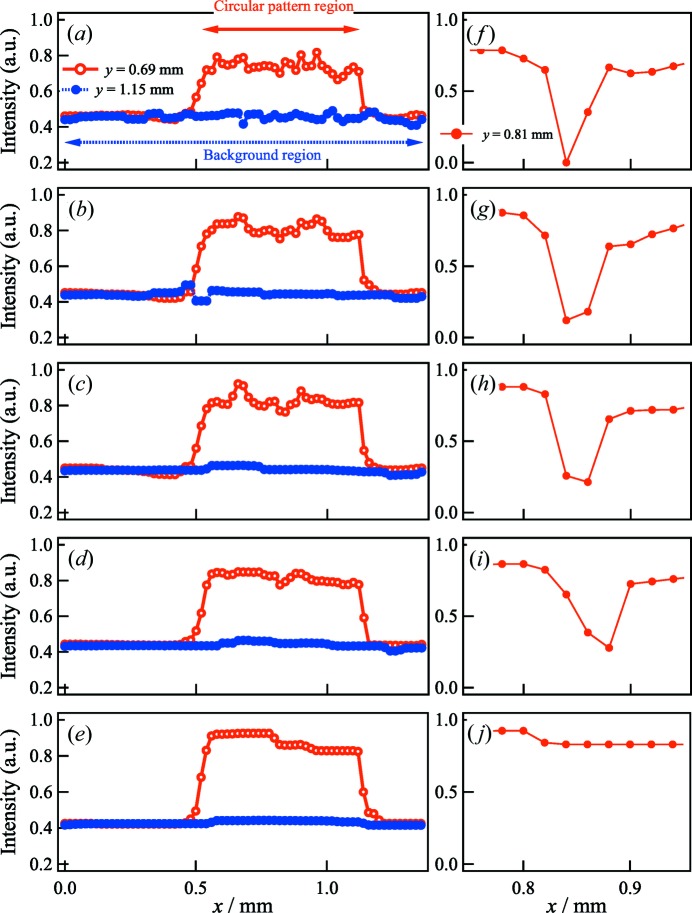
(Left) Cross-sectional normalized intensity profiles at *y* = 0.69 mm and *y* = 1.15 mm (along α and β) in Figs. 4 ▸(*g*)–4 ▸(*k*): (*a*) Δθ = 3.0°; (*b*) Δθ = 6.0°; (*c*) Δθ = 12.0°; (*d*) Δθ = 24.0°; and (*e*) Δθ = 48.0°. (Right) Cross-sectional normalized intensity profiles at *y* = 0.81 mm through A, F, K, P and S in Figs. 4 ▸(*g*)–4 ▸(*k*). (*f*) Δθ = 3.0°; (*g*) Δθ = 6.0°; (*h*) Δθ = 12.0°; (*i*) Δθ = 24.0°; and (*j*) Δθ = 48.0°.

Figs. 7 ▸(*a*)–7 ▸(*e*) show the cross-sectional normalized intensity profiles for *y* = 0.69 mm (α) and *y* = 1.15 mm (β) in Figs. 4 ▸(*g*)–4 ▸(*k*) as a function of *x*. Compared with the results shown in Fig. 5 ▸, TV regularization enhanced the signal intensities in the circular pattern and suppressed the noise in the background area. For interval angles of Δθ = 24.0° and Δθ = 48.0°, 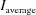 for the circular pattern drastically increased to 0.8. These profiles also indicated that the effect of smoothing for the noise and artifacts increased on the TV-CT image with larger interval angles. The profiles for *y* = 0.81 mm as a function of *x* indicated that the impurity was observable even at Δθ = 24.0° in Figs. 7 ▸(*f*)–7 ▸(*i*) [as indicated by A, F, K and P in Figs. 4 ▸(*g*)–4 ▸(*j*), respectively]. In the Δθ = 48.0° step, a flattened profile was obtained in the region of the impurity in Fig. 7 ▸(*j*) [as indicated by S in Fig. 4 ▸(*k*)], so it was difficult to identify this part.

To show how TV regularization improved the image quality for the signal and background areas quantitatively, MSE values as a function of Δθ were also plotted in Figs. 6 ▸(*a*) and 6 ▸(*b*) (as indicated by filled circles and squares). The results in Fig. 6 ▸(*a*) indicate that the estimated MSE values in the TV-CT images linearly increased as a function of Δθ. From Fig. 6 ▸(*a*), the MSE value of the circular pattern in the TV-CT image for the Δθ = 3.0° step was estimated to be 0.0014. In comparison with the FBP-CT image for the Δθ = 3.0° step, the value was approximately reduced by 46% in Fig. 6 ▸(*a*). For the case of Δθ = 6.0°, the MSE value increased to 0.0015; however, this value was lower than that derived from the true image of the Δθ = 1.0° step. In the case of Δθ = 12.0°, we achieved a reduction of 80% in the estimated MSE value. Notably, the estimated MSE value of 0.0017 was close to that from the FBP-CT image for Δθ = 3.0°. When the value of Δθ increased for 12.0 < Δθ ≤ 48.0°, the estimated MSE values continued to increase. As the interval angle increased, the smoothing effect on TV regularization depended on the magnitude of noise. On the other hand, the estimated MSE value from the FBP-CT images was almost constant for Δθ > 12.0°. As a result, in comparison with the MSE value from the FBP-CT image, the reduction in the MSE value from the TV-CT images decreased from 74 to 61% for 24.0 ≤ Δθ ≤ 48.0°. These results indicate that TV regularization provided the highest improvement in the downsampling images for Δθ = 12.0°. However, for the overall recovered images, the signal intensities were enhanced and the edges were preserved using TV regularization. This method also provided smoothing or signals with low noise.

In Fig. 6 ▸(*b*), we describe the results of using TV regularization for denoising in background areas. In the FBP-CT image, since the noise increased for higher interval angles, the estimated MSE value linearly increased for 3.0 ≤ Δθ ≤ 12.0°. For Δθ > 12.0°, the effect of ramp filtering could reduce noise; hence, the MSE values were almost constant for Δθ > 12.0°. When we estimated the MSE values from the TV-CT images, these values were more highly suppressed than those for the circular part in the FBP-CT images. For the MSE value of the Δθ = 3.0° step, the value decreased by approximately 21% from 0.0013 to 2.7 × 10^−4^. Compared with the MSE value from the FBP-CT image for the Δθ = 12.0° step, the value from the TV-CT images was reduced approximately by an order of magnitude. Overall for the TV-CT images, we obtained the result that the estimated MSE values from the TV-CT images were lower than those from the highest spatial resolution of the Δθ = 1.0° step from the FBP-CT images. This result indicated that the regularization parameter demonstrated a smoothing effect and was remarkably effective in the background areas.

## 4. Conclusions   

We investigated how TV regularization improves GISAXS-CT images with sparse Δθ values from 3 to 48°. GISAXS-CT measurements were performed to visualize the spatial distribution of nanostructures in circular patterned Au thin films. In comparison with the FBP-CT images, the image quality improved in the TV-CT images for all interval angles. In particular, the MSE values of the signal areas in the TV-CT images for Δθ < 12.0° were equal to or less than the value in the FBP-CT image for Δθ = 1.0°. Using TV regularization, we succeeded in improving the efficiency of data acquisition by at least a factor of 12 (Δθ < 12.0°). However, the MSE values in the TV-CT images for Δθ > 12.0° were higher than those for Δθ = 1.0° in the FBP-CT image because the circular pattern was blurred with higher noise in the signal areas. For the background areas, this algorithm significantly suppressed noise for all interval angles. The MSE values of the background areas in the TV-CT images were less than those in the FBP-CT image for Δθ = 1.0°.
